# Fatigue in the general population- associations to age, sex, socioeconomic status, physical activity, sitting time and self-rated health: the northern Sweden MONICA study 2014

**DOI:** 10.1186/s12889-017-4623-y

**Published:** 2017-08-14

**Authors:** Isak Engberg, Johan Segerstedt, Göran Waller, Patrik Wennberg, Mats Eliasson

**Affiliations:** 1 Department of Public Health and Clinical Medicine, Sunderby Research Unit, Umeå University, Umeå, Sweden; 2 Department of Public Health and Clinical Medicine, Family Medicine, Umeå University, Umeå, Sweden; 3 Department of Medicine, Sunderby Hospital, 971 80 Luleå, Sweden

## Abstract

**Background:**

Fatigue is widespread in the population and a common complaint in primary care. Little is known about prevalence of fatigue in the population and its predictors.

We aimed to describe the pattern of fatigue in the general population and to explore the associations with age, sex, socioeconomic status, self-reported physical activity, sitting time and self-rated health.

**Methods:**

One thousand, five hundred and fifty-seven out of 2500 invited subjects in the Northern Sweden MONICA Study 2014, aged 25–74 years, filled out the Multidimensional Fatigue Inventory (MFI-20), consisting of four subscales: General fatigue (GF), Physical fatigue (PF), Reduced activity (RA) and Mental fatigue (MF). Questions regarding age, sex, socioeconomic status, physical activity, sitting time and self-rated health were also included.

**Results:**

Higher age correlated significantly with lower fatigue scores for the GF and MF subscales. Women had higher fatigue scores than men on all subscales (*p* < 0.05). Among men, higher socioeconomic status was related to lower fatigue for the GF, PF and RA subscales (age adjusted *p < 0.05).* Among women, higher socioeconomic status was related to lower fatigue for the PF and MF subscales (age adjusted *p* < 0.05)*.* Higher physical activity was connected to lower levels of fatigue for all subscales (age and sex adjusted *p* < 0.001) except for MF. Longer time spent sitting was also related to more fatigue on all subscales (age and sex adjusted *p < 0.005)* except for MF. Better self-rated health was strongly associated with lower fatigue for all subscales (age and sex adjusted *p* < 0.001).

**Conclusion:**

Older, highly educated, physically active men, with little sedentary behavior are generally the least fatigued. Self-rated health is strongly related to fatigue. Interventions increasing physical exercise and reducing sedentary behavior may be important to help patients with fatigue and should be investigated in prospective studies.

## Background

Fatigue is widespread in the population [1–4], it is frequently reported in primary care [5, 6], and it is commonly unexplained by underlying disease [6, 7]. Research about prevalence of fatigue in the general population is sparse and uses a broad range of methods to measure fatigue.

The concept of fatigue is multidimensional and lacking a universally accepted definition. A systematic review of instruments for measuring fatigue has proposed one definition: “An unpleasant physical, cognitive and emotional symptom described as a tiredness not relieved by common strategies that restore energy. Fatigue varies in duration and intensity and it reduces, to different degrees, the ability to perform the usual daily activities.” [8].

Fatigue is also a common and debilitating symptom in many diseases such as cancer, neurological diseases, stroke and depression [2, 3, 7, 9]. A recent paper from the EPIC-Norfolk study has even linked fatigue in the general population to excess mortality [10]. We have previously showed that patients with type 1 diabetes experience more fatigue than the general population [11].

Although the concept of fatigue has been explored among different groups of patients, only a few studies have examined the distribution of fatigue in the general population using well validated scales [2, 4, 10, 12]. More fatigue has been reported in association with advancing age [2, 4], female sex [2, 4, 10, 12] and lower socioeconomic status [4]. Commonly, these studies utilize hospital staff, military recruits and similar highly selected groups as their reference population [3, 13].

Physical activity is related to fatigue. Graded exercise therapy has proven useful in the treatment for some forms of chronic fatigue syndrome [14, 15]. Low physical activity is more common in fatigued patients with chronic obstructive pulmonary disease [16]. Fatigue also affects physical function [1]. Presumably regular physical activity could have positive effects on fatigue in general, not only in chronic fatigue syndrome, although the causality direction is unclear. Recent studies indicate that time spent sedentary may be a crucial factor for fatigue [17, 18], but this has not been shown in large, population based studies.

The concept of self-rated health integrates many domains of health and disease, predicts many health outcomes and relates to markers of socioeconomic vulnerability. It is even associated with stroke and myocardial infarction [19]. The relationship between self-rated health and fatigue has not been studied using validated methods, but one paper has explored variables related to self-rated health and indicates that there is an association [20].

One test developed to assess fatigue is the Multidimensional Fatigue Inventory (MFI-20) [13]. The psychometric properties have been tested in many patient groups and among healthy volunteers. There is a Swedish version, and the test is valid and reliable [1–3, 8, 12].

Our primary aim was to analyze the pattern of fatigue in the general population, using MFI-20, focusing on the effects of age and sex but also considering socioeconomic status. Secondary aims were to explore if fatigue is associated with physical activity, sitting time or self-rated health.

## Methods

### MFI-20

MFI-20 consists of five subscales regarding fatigue: General fatigue (GF), Physical fatigue (PF), Reduced motivation (RM), Reduced activity (RA) and Mental fatigue (MF) [13]. The questionnaire consists of 20 items for which the person fills out, on a five-point Likert scale, to what extent the statement is true for him/her. “Yes, that is true” constitutes one end and “No, that is not true” makes up the other end of the scale. Each subscale ranges from 4 to 20 points and higher scores indicate a higher level of fatigue. A recent study reports that the subscale RM is considered to be in the range of weak scalability and therefore the results from this subscale should be interpreted with caution [12]. We excluded this subscale.

### Study population

Data from the 2014 population study of the Northern Sweden MONICA population survey was used [11, 21]. Subjects, 25–74 years of age, were randomly selected from continuously updated population registries and stratified by sex and 10 year age-groups. No exclusion criteria were applied. Details of selection and sampling have been described previously [21]. One thousand, five hundred and fifty-seven participants out of 2500 invited (62%), filled out the MFI-20 form along with basic questions regarding age, sex, socioeconomic status, physical activity, sitting time, and self-rated health [11, 21].

### Procedure

The highest attained education was used as a proxy for socioeconomic status and dichotomized into university education (yes/no).

Physical activity was quantified using the Cambridge physical activity index, a validated method to calculate total physical activity [22]. The Cambridge index consists of two domains, one regarding physical activity during work and the other regarding physical activity during leisure time. In cases where the Cambridge index could not be calculated (*n* = 499), mostly due to missing data regarding physical activity during work, the following question was used as a substitute: “How much have you moved about and exerted yourself physically in your leisure time during the past 12 months” with four alternatives: “Sedentary during leisure time”, “Moderate exercise during leisure time”, “Moderate but regular exercise during leisure time”, and “Regular exercise and training during leisure time”.

To assess sedentary time, the question “How much time do you spend sitting during a usual day?” was used. Self-reported sitting time was then categorized into <4.5 h, 4.5–7.5 h and >7.5 h. Previous reports regarding sitting time, with data from the Eurobarometer surveys, used these categories [23].

Self-rated health was measured on a three-grade ordinal scale by the question “How would you assess your general health condition compared to persons of your own age?” with the alternatives “better”, “similar” or “worse” [19].

### Statistical analysis

Differences in fatigue level in relation to age, sex and other variables described above were investigated by comparing mean scores for each subscale, using independent samples *t*-test for variables consisting of two categories or One-way ANOVA for variables consisting of more than two categories. A univariate General Linear Model (GLM) was used to calculate the associations taking multiple variables such as age, sex and education into account. GLM regression coefficient – β, was used to calculate the impact of age on fatigue scores. 95% confidence intervals (CI) are reported.

Participants who did not reply to a certain question were excluded from that analysis but included in other analyses that did not depend on that missing variable. None of the variables, except the Cambridge index (missing in 32.3%) and self-reported sitting time (missing in 6.5%) had a higher missing rate than 2.5%. The Cambridge index was missing 499 values, mainly because of participants who did not report their physical activity during work.

All statistical calculations were done with SPSS 24.0 for Windows.

## Results

### Study population

A total of 1557 out of 2500 invited participated, 51.7% women and 48.3% men. Distribution of background variables are shown in Table 1.

**Table 1:** Basic characteristics for the study population

	Men *n* = 747 (48.3%)	Women *n* = 798 (51.7%)
Age (years), mean	52	51
Age-groups (%)		
25–34 y	13.8	14.2
35–44 y	16.9	19.3
45–54 y	21.3	21.4
55–64 y	22.9	22.4
65–74 y	25.2	22.7
Self-rated health (%)		
Better than peers	26.8	21.4
Similar	60.8	60.6
Worse than peers	12.3	18.0
University education (%)		
Non-university education	74.0	60.6
University education	26.0	39.4
Cambridge physical activity index (%)		
Inactive	17.7	14.8
Moderately inactive	11.5	12.7
Moderately active	19.4	17.5
Active	20.2	21.7
Missing Cambridge index data	31.2	33.3
Self-reported sitting-time (%)		
<4.5 h	34.5	39.9
4.5–7.5 h	32.2	35.1
>7.5 h	33.2	25.0

### Sex and age

**Fig. 1:**
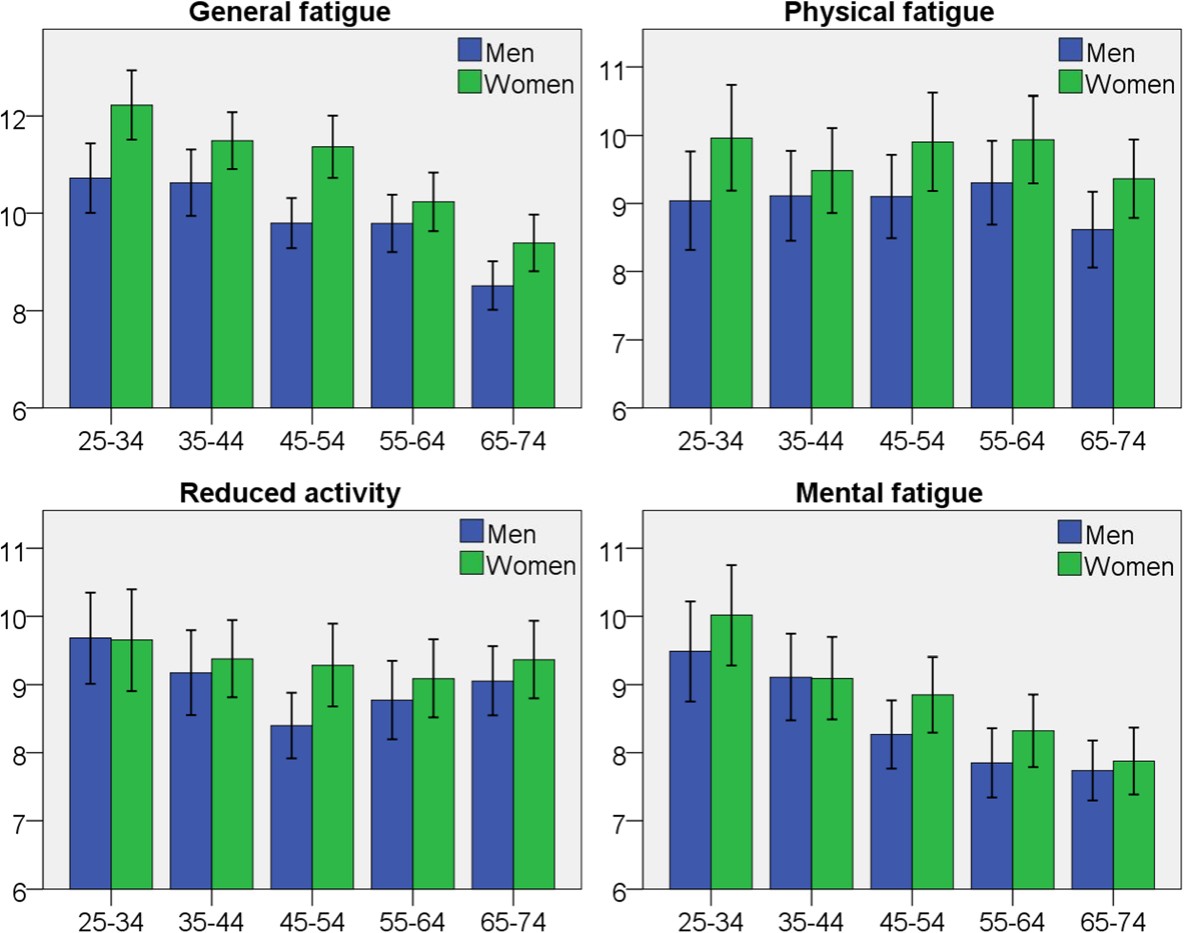
Bar charts for each subscale of fatigue according to age-groups (years). Y-axis with mean fatigue score. Error bars 95% CI

Fatigue scores according to sex and age group are presented in Fig. 1 and Table 2. Women had significantly higher mean fatigue scores than men on all four subscales (*p* < 0.05) and in most age groups, most markedly in GF and PF and with less difference in RA and MF.

**Table 2:** Fatigue scores for the subscales of MFI-20, stratified by sex and age groups

General fatigue				Physical fatigue		
Age	Men	Women	*p*-value	Men	Women	*p*-value
25–34 y	10.7	12.2	0.004	9.0	10.0	0.087
35–44 y	10.6	11.5	0.056	9.1	9.5	0.4
45–54 y	9.8	11.4	<0.001	9.1	9.9	0.097
55–64 y	9.8	10.2	0.3	9.3	9.9	0.2
65–75 y	8.5	9.4	0.025	8.6	9.4	0.067
Total	9.7	10.8	<0.001	9.0	9.7	0.001
Reduced activity				Mental fatigue		
25–34 y	9.7	9.7	1	9.5	10.0	0.3
35–44 y	9.2	9.4	0.6	9.1	9.1	1
45–54 y	8.4	9.3	0.026	8.3	8.9	0.1
55–64 y	8.8	9.1	0.4	7.9	8.3	0.2
65–75 y	9.1	9.4	0.4	7.7	7.9	0.7
Total	9.0	9.3	0.046	8.4	8.7	0.044

Fatigue scores were generally lower in higher aged subjects, with Pearson correlation coefficients ranging between −0.229 and −0.032. However, this negative correlation between fatigue and age was only significant for GF and MF. The regression coefficient (β), for age on GF was −0.065 (CI -0.079 – -0.051) (*p* < 0.001) and for MF it was −0.049 (CI -0.061 – -0.036) (*p* < 0.001). This means that for each year of increasing age, GF mean scores decreased by 0.065 and MF mean scores decreased by 0.049. For PF and RA, the coefficient was negative but non-significant. The pattern was similar in both men and women.

### Socioeconomic status

**Fig. 2:**
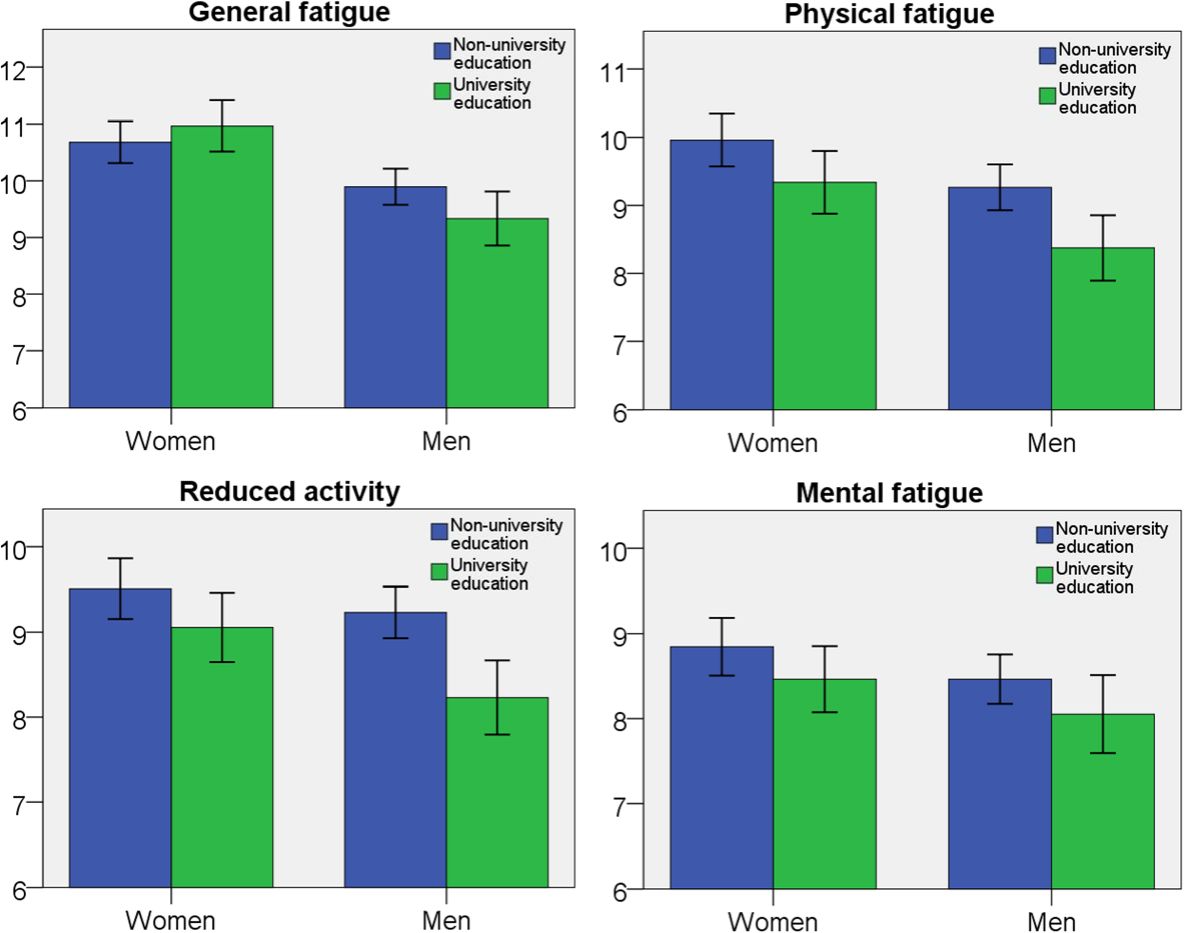
Bar charts for each subscale of fatigue in relation to educational level. Y-axis with mean fatigue score. Error bars 95% CI

Among men university education was significantly associated with lower mean fatigue scores for the PF and RA subscales. Among women, lower mean scores for PF were significantly associated with having a university education (Fig. 2). Among men, after adjustment for age, lower scores for the GF, PF*,* and RA subscales were associated with university education (*p* < 0.05), MF was not*.* Among women, after adjustment for age, lower PF and MF scores were associated with having a university education (*p* < 0.05) but GF and RA were not.

### Physical activity

**Fig. 3:**
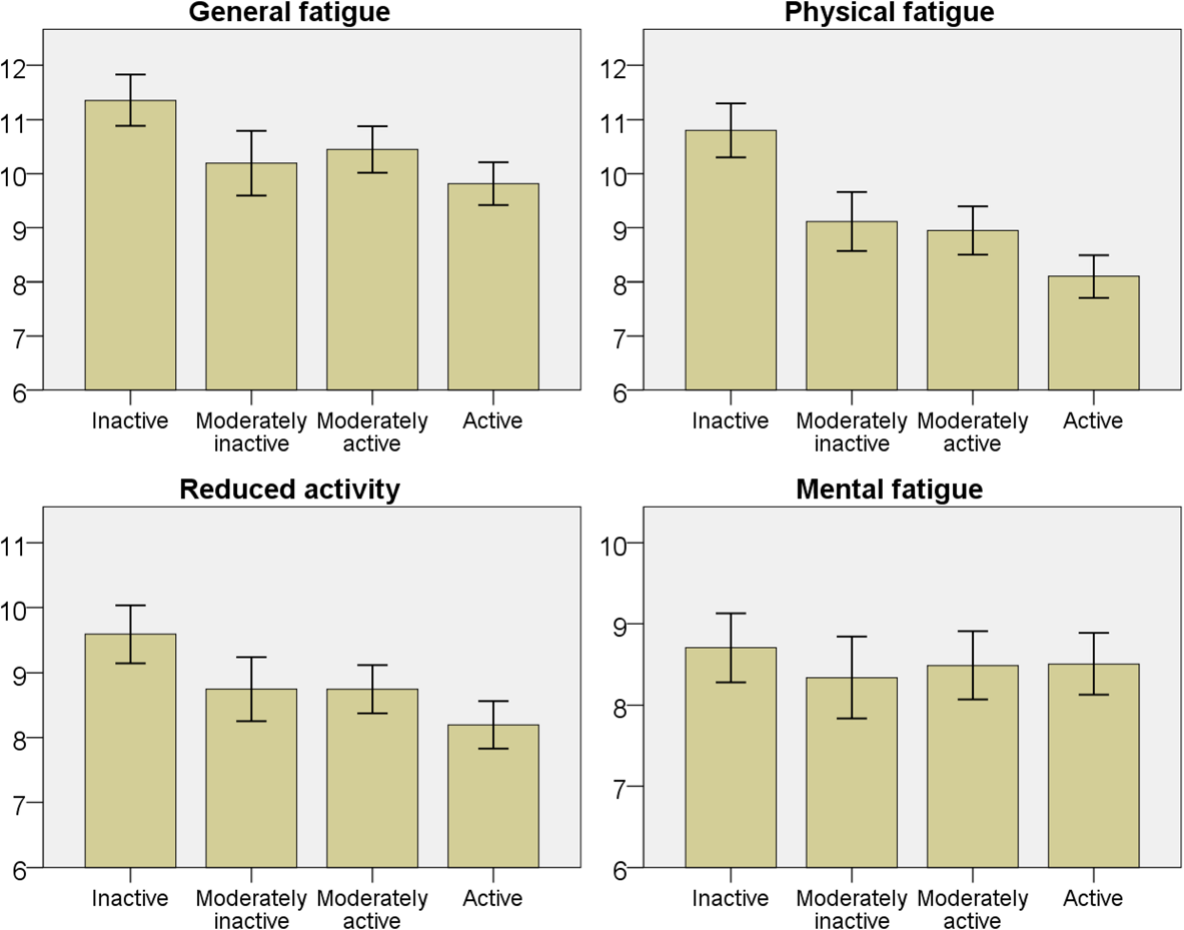
Bar charts for each subscale of fatigue in relation to physical activity measured by Cambridge physical activity index. Y-axis with mean fatigue score. Error bars 95% CI

Using the Cambridge index classification of activity-level, higher activity-level was associated with lower mean fatigue scores for three subscales (*p* < 0.001) but not for MF (Fig. 3). After adjustment for age, sex and socioeconomic status, the associations remained highly significant (*p* < 0.001).

**Fig. 4:**
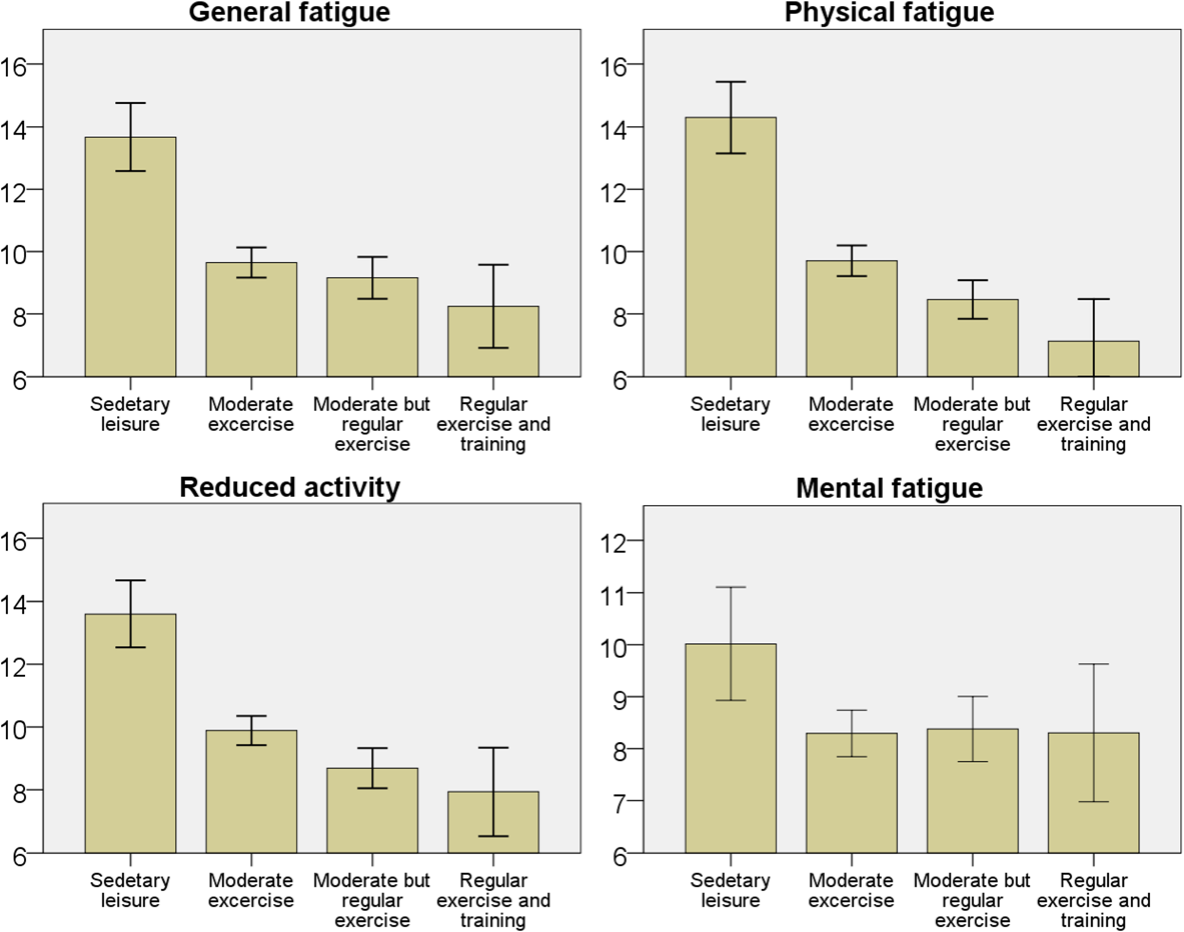
Bar charts for each subscale of fatigue in relation to physical activity during leisure time for the past 12 months (for individuals with missing data for the Cambridge physical activity index). Y-axis with mean fatigue score. Error bars 95% CI

For participants not classified by the Cambridge index, higher physical activity during leisure time was associated with lower fatigue on all subscales (*p* < 0.01) (Fig. 4). After adjustment for age, sex and socioeconomic status, the associations remained highly significant (*p* < 0.001) for all subscales except for MF*.*

### Sitting time

**Fig. 5:**
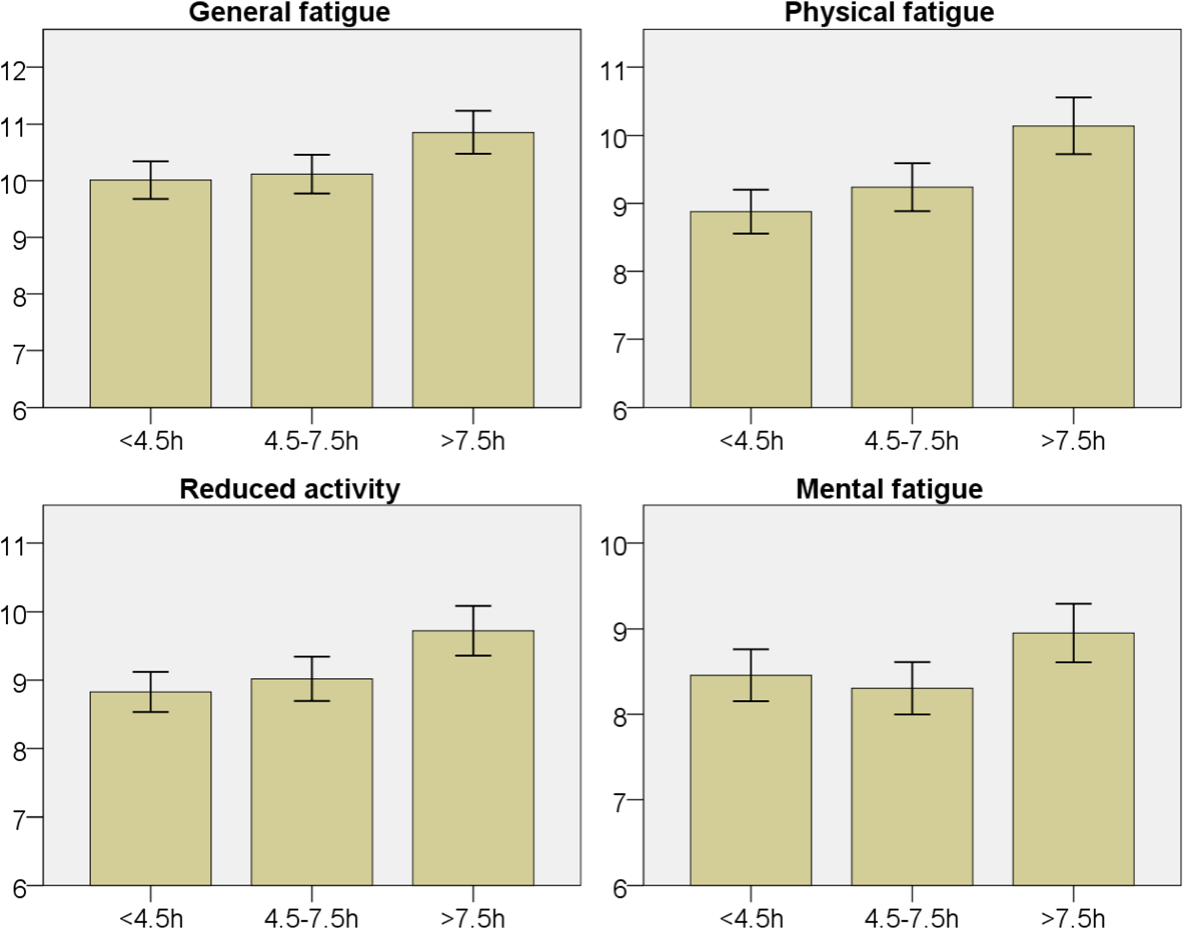
Bar charts for each subscale of fatigue in relation to self-reported sitting time (hours). Y-axis with mean fatigue score. Error bars 95% CI

Longer self-reported sitting time was associated with higher mean fatigue scores on all fatigue subscales (*p* < 0.005 for GF*,* PF and RA; *p <* 0.05 for MF) (Fig. 5). After adjustment for age, sex and socioeconomic status, MF was no longer significantly associated with self-reported sitting time, but the association remained strong for the other subscales.

### Self-rated health

**Fig. 6:**
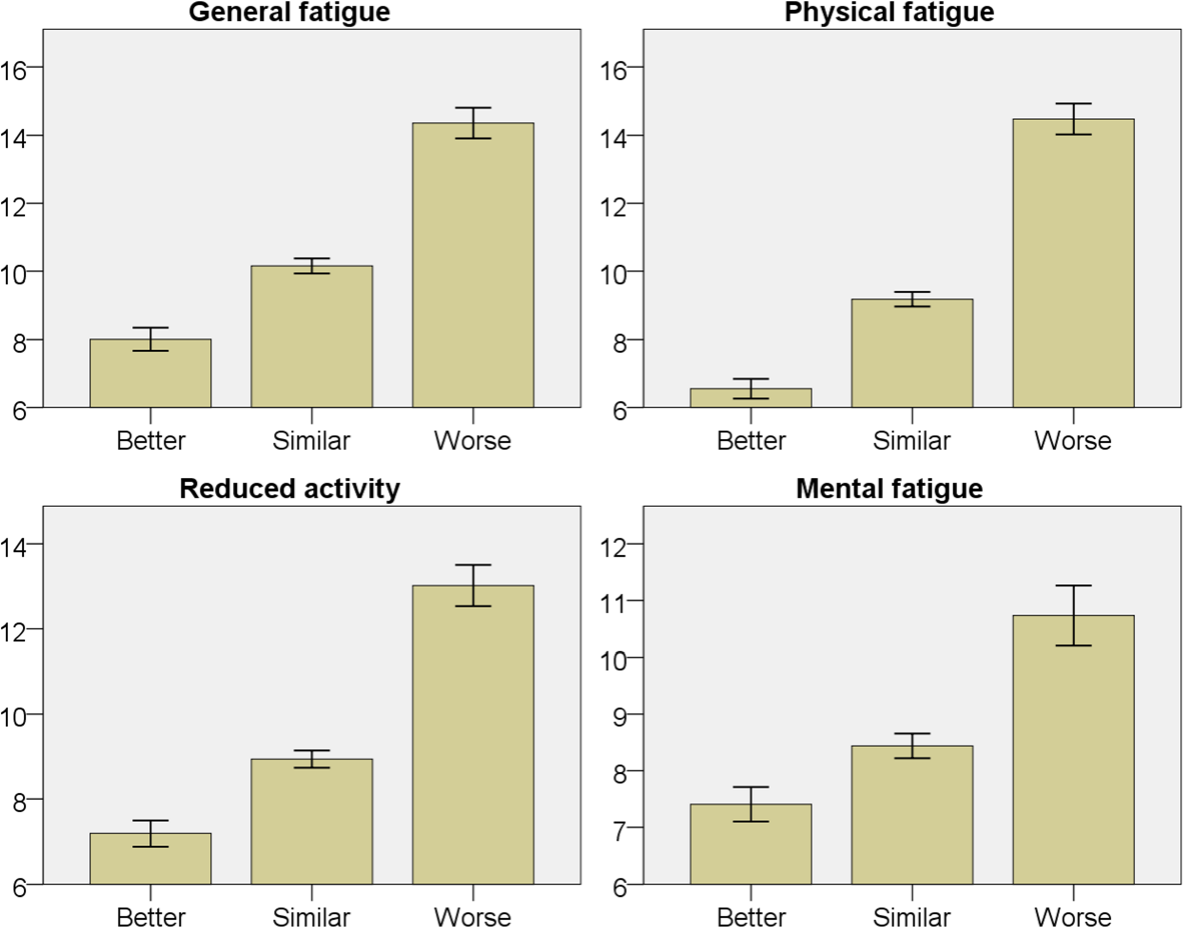
Bar charts displaying each subscale of fatigue in relation to self-rated health. Y-axis with mean fatigue score. Error bars 95% CI

Better self-rated health was strongly associated with lower mean fatigue scores for all subscales (*p* < 0.001) (Fig. 6). Mean difference in fatigue scores between lowest self-rated health and highest were 6.4 (CI 5.8–6.9) for GF, 7.9 (CI 7.4–8.4) for PF, 5.8 (CI 5.3–6.4) for RA and 3.3 (CI 2.8–3.9) for MF. After adjustment for age, sex and socioeconomic status, self-rated health remained a highly significant predictor for all fatigue subscales (*p* < 0.001).

## Discussion

Our study shows that fatigue is associated with female sex as well as with lower age. Physically active people have lower levels of fatigue and sedentary people experience more fatigue. University-educated people have lower levels of fatigue. Lastly, having a poor perspective of one’s health seems strongly related to fatigue.

### Sex, age and socioeconomic status

Earlier studies regarding fatigue prevalence have reported that women have higher fatigue levels than men [2, 4, 10, 12], and that lower socioeconomic status relates to more fatigue [4]. Our study confirms those findings. In contrast to our present results, earlier studies generally report more fatigue with advancing age, rather than less [2, 4].

It has been known for a long time that gender and social class are related to many health inequities, such as differences in life expectancy [24]. Fatigue is one such inequity. Many diseases follow a social gradient. Gender and social class interact closely and lead to differences in distribution of resources. Perhaps fatigue is a bodily expression of ill-being, which is related to other health inequities, economic factors and unequal assets in life. Fatigue might enhance health inequities further, since women in the lowest socioeconomic class are more prone to feel fatigued and therefor might be less likely to be physically active. This could further increase the ill-being for these individuals and the social discrimination of this group. The gap in general fatigue between men and women was largest among those below 55 years of age which indicates that factors related to gender inequalities regarding household responsibilities and child raising may be responsible.

As stated above, our findings regarding how age impacts on fatigue partly contradict earlier papers. We did not take into account any diseases such as cancer, psychiatric- or cardiovascular diseases when analyzing our data, but this would probably just enhance age influence on fatigue, since the disease burden in general increases with advancing age. If we view fatigue as a bodily expression of one’s health and well-being, then our findings could be explained by the relatively economically stable, healthy and unstressful life of elderly people in Sweden [25]. But declining fatigue with higher age, especially general and mental, was linear across ages, without any threshold effect after retirement which point to unmeasured causal factors other than retirement.

### Physical activity and sedentary behavior

As stated earlier, graded exercise therapy has been proven effective in treatment for some cases of chronic fatigue syndrome [14, 15]. We now show that fatigue in the general population relates to a measure of total physical activity level and also to physical activity during leisure time.

The causality is most likely bidirectional. Physical activity is essential for treating and preventing many somatic and psychiatric diseases. In essence, the human being is made for, and needs physical activity. The lack of physical activity can partly express itself as fatigue. On the other hand, a fatigued person is probably less likely to be physically active for the apparent reason that fatigue negatively influences vitality and motivation. This negative loop may enhance itself, and an intervention to reduce fatigue might be to break this loop by encouraging physical activity.

Sedentary behavior, which is distinct from physical inactivity [26], has previously been linked to excess fatigue in smaller studies [17, 18]. We can now show that in this large, population-based study. This should be investigated further in prospective studies.

### Self-rated health

The association between fatigue and self-rated health has not been well addressed in previous research. Low self-rated health has been found to be associated with increased levels of interleukin 6 and CRP, which is involved in inflammatory responses [27]. Low self-rated health has been associated with increased physiologic stress response, in order to maintain stability (allostasis) [28]. Thus, there are several plausible pathways by which self-rated health could affect the body. Fatigue may be a consequence of these biological responses or may cause the low self-rated health. A paper from 2009 proposed that self-rated health was influenced by bodily sensations [29]. Fatigue could be such a sensation. The association between fatigue and self-rated health is also interesting, as this could imply that interventions to improve self-rated health, could do so by reducing fatigue.

Minimal clinical important differences (MCID), defined as the smallest change in an outcome that a patient would identify as important, is a tool used to distinguish between statistically significant values and clinically important differences. MCID varies for MFI-20 in different studies and is dependent on the method used to calculate MCID [30]. In a radiotherapy population MCID was calculated for MFI-20, and this yielded values between −1.91 (PF) and −3.27 (MF), with GF and RA in between [31]. As most of the differences in MFI-20 seen between categories of leisure time physical activity, sedentary behaviour and self-rated health are of this magnitude we claim them to be clinically important.

### Implications

Fatigue should be viewed as a continuum, related to many factors in life, only some of which have been explored in this paper. Possibly improving these factors could lower fatigue levels, but lowering fatigue levels might positively influence these factors as well. For example, lower fatigue could probably increase one’s motivation for physical activity during leisure time, but being physically active will likely lower fatigue levels.

Doctors already know that encouraging physical activity and avoiding sedentary behavior is important for diseases such as diabetes mellitus, cardiovascular illnesses and depression. Our findings could support the idea that physical activity might help reduce fatigue as well.

Both fatigue and physical inactivity has been associated with inflammatory markers such as cytokines although causality is not proven. Thus, an ongoing randomized study on physical activity in cancer patients is of great interest as both fatigue and cytokines will be evaluated [32].

We aimed to describe the pattern of fatigue in the general population. We have provided a reference table, divided by sex and age-groups for each subscale of fatigue from MFI-20. This could be used as a reference population together with MFI-20 forms in clinical practice when patients seek consultation regarding fatigue and when doctors are assessing specific diseases. Clinical studies today often use patient related outcome measures (PROMs). EQ-5D and RAND-36/SF-36 are the standard choice for these studies. Possibly fatigue, assessed with MFI-20, and in combination with our reference population, could be used as a PROM in future studies. The validity, relevance and test properties for MFI-20 have been reported and found satisfactory [3, 13].

### Strengths and limitations of the study

The strength of this study lies in the use of the recent Northern Sweden MONICA Study population sample, which gives our findings a strong external validity for the Swedish society but of uncertain validity in other countries. However, the material has one major drawback: in the youngest age group, 25–34 years, the participation rate (43%) was considerably lower than in the other age-groups. The pattern of fatigue in the different age groups although remained similar throughout the whole cohort. Both MFI-20 [3, 8, 12] and the Cambridge index [22] have been validated thoroughly, which yields high internal validity for our study. One variable, related to physical activity during work, which is part of the Cambridge index, had a high number of missing values. This fact was interpreted as responses from subjects who were not working because 79% of participants who failed to respond to that question were in the oldest two age groups (55–64 and 65–74 y). This is a cross-sectional study; therefor it is limited in regards to determine causality, we can only speculate.

## Conclusion

Older, highly educated, physically active men, with little sedentary behavior, are generally the least fatigued. Self-rated health is strongly related to fatigue, and the connection between the two should be investigated more closely in future research. Interventions regarding physical exercise and reducing sedentary behavior may be important to help patients with fatigue and should be investigated in prospective studies.

## References

[1] Boter H, Manty M, Hansen AM, Hortobagyi T, Avlund K (2014). Self-reported fatigue and physical function in late mid-life. J Rehabil Med.

[2] Schwarz R, Krauss O, Hinz A (2003). Fatigue in the general population. Onkologie.

[3] Lundh Hagelin C, Wengström Y, Runesdotter S, Johan Fürst C (2007). The psychometric properties of the Swedish multidimensional fatigue inventory MFI-20 in four different populations. Acta Oncol.

[4] Watt T, Groenvold M, Bjorner JB, Noerholm V, Rasmussen NA, Bech P (2000). Fatigue in the Danish general population. Influence of sociodemographic factors and disease. J Epidemiol Community Health.

[5] Cullen W, Kearney Y, Bury G (2002). Prevalence of fatigue in general practice. Ir J Med Sci.

[6] Ridsdale L, Evans A, Jerrett W, Mandalia S, Osler K, Vora H (1993). Patients with fatigue in general practice: a prospective study. BMJ.

[7] Nijrolder I, van der Windt D, de Vries H, van der Horst H (2009). Diagnoses during follow-up of patients presenting with fatigue in primary care. CMAJ.

[8] Mota DD, Pimenta CA (2006). Self-report instruments for fatigue assessment: a systematic review. Res Theory Nurs Pract.

[9] MacKean PR, Stewart M, Maddocks HL (2016). Psychosocial diagnoses occurring after patients present with fatigue. Can Fam Physician.

[10] Basu N, Yang X, Luben RN, Whibley D, Macfarlane GJ, Wareham NJ, Khaw K, Myint PK (2016). Fatigue is associated with excess mortality in the general population: results from the EPIC-Norfolk study. BMC Med.

[11] Segerstedt J, Lundqvist R, Eliasson M (2015). Patients with type 1 diabetes in Sweden experience more fatigue than the general population. J Clin Transl Endocrin.

[12] Fieo RA, Mortensen EL, Lund R, Avlund K (2014). Assessing fatigue in late-midlife: increased scrutiny of the multiple fatigue inventory-20 for community-dwelling subjects. Assessment.

[13] Smets E, Garssen B (1995). Bonke Bd, de Haes J: the multidimensional fatigue inventory (MFI) psychometric qualities of an instrument to assess fatigue. J Psychosom Res.

[14] Castell BD, Kazantzis N, Moss-Morris RE (2011). Cognitive behavioral therapy and graded exercise for chronic fatigue syndrome: a meta-analysis. Clin Psychol Sci Pract.

[15] Larun L, Brurberg K, Odgaard-Jensen J, Price JR. Exercise therapy for chronic fatigue syndrome. Cochrane Database Syst Rev. 2017;(4):CD003200. doi:10.1002/14651858.CD003200.pub7. 10.1002/14651858.CD003200.pub7 PMC6419524 28444695

[16] Andersson M, Stridsman C, Rönmark E, Lindberg A, Emtner M (2015). Physical activity and fatigue in chronic obstructive pulmonary disease–a population based study. Respir Med.

[17] Wennberg P, Boraxbekk CJ, Wheeler M, Howard B, Dempsey PC, Lambert G, Eikelis N, Larsen R, Sethi P, Occleston J, Hernestal-Boman J, Ellis KA, Owen N, Dunstan DW (2016). Acute effects of breaking up prolonged sitting on fatigue and cognition: a pilot study. BMJ Open.

[18] Ellingson LD, Kuffel AE, Vack NJ, Cook DB (2014). Active and sedentary behaviors influence feelings of energy and fatigue in women. Med Sci Sports Exerc.

[19] Waller G, Janlert U, Hamberg K, Forssen A (2016). What does age-comparative self-rated health measure? A cross-sectional study from the northern Sweden MONICA project. Scand J Public Health.

[20] Taloyan M, Leineweber C, Hyde M, Westerlund H (2015). Self-rated health amongst male and female employees in Sweden: a nationally representative study. Int Arch Occup Environ Health.

[21] Eriksson M, Forslund AS, Jansson JH, Soderberg S, Wennberg M, Eliasson M (2016). Greater decreases in cholesterol levels among individuals with high cardiovascular risk than among the general population: the northern Sweden MONICA study 1994 to 2014. Eur Heart J.

[22] InterAct Consortium (2012). Validity of a short questionnaire to assess physical activity in 10 European countries. Eur J Epidemiol.

[23] Milton K, Gale J, Stamatakis E, Bauman A (2015). Trends in prolonged sitting time among European adults: 27 country analysis. Prev Med.

[24] Novak M. Social inequity in health: Explanation from a life course and gender perspective (Thesis). Umeå: Department of Public Health & Clinical Medicine, Family Medicine, Umeå University; 2010.

[25] The Global AgeWatch Index 2015. http://www.helpage.org/global-agewatch/. Accessed 7 Dec 2016.

[26] Ekelund U, Steene-Johannessen J, Brown WJ, Fagerland MW, Owen N, Powell KE, Bauman A, Lee I, Series LPA (2016). Lancet sedentary behaviour working group: does physical activity attenuate, or even eliminate, the detrimental association of sitting time with mortality? A harmonised meta-analysis of data from more than 1 million men and women. Lancet.

[27] Christian LM, Glaser R, Porter K, Malarkey WB, Beversdorf D, Kiecolt-Glaser JK (2011). Poorer self-rated health is associated with elevated inflammatory markers among older adults. Psychoneuroendocrinology.

[28] Hasson D, Von Thiele SU, Lindfors P (2009). Self-rated health and allostatic load in women working in two occupational sectors. J Health Psychol.

[29] Jylhä M (2009). What is self-rated health and why does it predict mortality? Towards a unified conceptual model. Soc Sci Med.

[30] Goligher EC, Pouchot J, Brant R, Kherani RB, Avina-Zubieta JA, Lacaille D, Lehman AJ, Ensworth S, Kopec J, Esdaile JM, Liang MH (2008). Minimal clinically important difference for 7 measures of fatigue in patients with systemic lupus erythematosus. J Rheumatol.

[31] Purcell A, Fleming J, Bennett S, Burmeister B, Haines T (2010). Determining the minimal clinically important difference criteria for the multidimensional fatigue inventory in a radiotherapy population. Support Care Cancer.

[32] Berntsen S, Aaronson NK, Buffart L, Börjeson S, Demmelmaier I, Hellbom M, Hojman P, Igelström H, Johansson B, Pingel R, Raastad T, Velikova G, Åsenlöf P, Nordin K (2017). Design of a randomized controlled trial of physical training and cancer (phys-can) - the impact of exercise intensity on cancer related fatigue, quality of life and disease outcome. BMC Cancer.

